# Psychometric properties of the Farsi version of the gaming disorder scale for adolescents (GADIS-A)

**DOI:** 10.1186/s40359-022-00899-1

**Published:** 2022-08-08

**Authors:** Ali Mazaherizadeh, Zahra Taherifar, Hojjatollah Farahani

**Affiliations:** 1 Department of Psychology, Faculty of Psychology and Educational Sciences, University of Tehran, Tehran, Iran; 2 Department of Psychology, Faculty of Humanities, Tarbiat Modares University, Tehran, Iran

**Keywords:** Gaming disorder scale, Internet gaming disorder, Adolescent, Validity, Reliability

## Abstract

**Background:**

Gaming disorder was added to the 11th version of the international classification of disease by the world health organization in early 2019. Adolescents are the most vulnerable group in this area. Thus, a screening tool for this age range is essential. This study aims to examine the psychometric properties of the gaming disorder scale for adolescents (GADIS-A) in an Iranian male sample.

**Methods:**

260 male students-7th to 12th grade-from Isfahan city in the academic year 2020–2021 were selected using convenience sampling. The participants responded to the Farsi version of the GADIS-A and problematic online game questionnaire (POGQ). Thirty participants answered the scale again to assess the validity of the retest. Pearson’s correlation analysis, Cronbach’s alpha, and confirmatory factor analysis were used. The data were analyzed by SPSS version 24 and R software packages psych and lavaan.

**Results:**

Confirmatory factor analysis revealed that the two-factor model, which included cognitive-behavioral symptoms and negative consequences, had good fitness indices. The GADIS-A convergent validity is confirmed by the scale’s significant correlation with the POGQ. Cronbach’s alpha coefficient was used to determine the scale’s validity, which was 0.85 for the full scale and 0.70 and 0.75 for two factors. The validity of the retest after two weeks also showed a correlation of 0.88.

**Conclusion:**

The Farsi version of the gaming disorder scale for adolescents has a two-factor structure and is valid for use in Iran.

## Introduction

People use digital games for many reasons: relaxation, challenge, social interaction, and recreation [1]. For most, gaming is an enjoyable activity that can improve social and cognitive skills [2] and is also helpful in teaching [3]. Although gaming has some benefits [4], gaming without limits can be addicting [5] and cause negative consequences [6]. As a result, Internet Gaming Disorder (IGD) was added to Section III of the DSM-5 as a diagnosis that needs further research in 2013 [7]. IGD consists of nine criteria that apply to online gaming or gaming on any electronic device: (1) preoccupation with gaming, (2) withdrawal when not playing, (3) tolerance, (4) unsuccessful attempts to reduce or stop gaming, (5) giving up other activities, (6) continued gaming despite problems, (7) deception or covering up gaming, (8) gaming to escape negative moods, and (9) risking or losing relationships or career opportunities as a result of excessive gaming [8].

In addition, Gaming Disorder (GD) was added to the 11th version of the International Classification of Diseases (ICD-11) by the World Health Organization in early 2019. The following three criteria must be present to diagnose GD: a consistent and recurrent pattern of gaming activity (digital or video games) offline or online; (1) inability to control the game, (2) prioritization of the game above other activities, and (3) continuation or escalation of the game despite negative consequences [9].

The criteria for this disorder are different in ICD-11 and DSM-5 [10, 11]. The ICD-11 framework, for example, highlights the functional impairment part of GD, which means GD Clinical symptoms should be severe enough to affect personal, family, social, educational, occupational, and/or other aspects of life [10]. While these negative consequences are merely one of the nine DSM criteria, they are not necessary for diagnosis. Moreover, the DSM-5 framework, on the other hand, includes an extensive range of cognitive and behavioral symptoms of the disorder [7]. Furthermore, The WHO has set exclusion criteria for diagnosing GD in the ICD-11. These include hazardous gaming, bipolar type I, and bipolar type II [9].

On the other hand, children and adolescents are more susceptible to GD due to immaturity and limited cognitive capacity [12–14]. The most prevalent concerns among GD adolescents include sleep disorders, unsatisfactory school grades, family conflicts, and emotional and behavioral problems [15]. According to neuroimaging findings, adolescents with GD and those with drug use disorders have similar brain functions [16, 17]. Adolescent gaming alters the brain [18–20]. For example, research indicates; Prefrontal cortex instability leads to cognitive control, temporoparietal changes may affect attentional problems, and frontolimbic regions appear to be linked to poor emotional regulation and impaired emotional reactivity [21].

Epidemiological studies indicated adolescents have a higher prevalence rate of GD [22, 23]. GD prevalence among children and adolescents in different countries and samples is reported in the range of 2 to 13% [24, 25]. Recently Kim et al. [26] indicated that the pooled prevalence of GD is 3.3%, close to Stevens et al. [27] meta-analysis estimate, which is 3.05%. Both studies report that the GD prevalence in boys is 2.5 times higher than in girls. It is important to note that being male is one of the risk factors for GD [28, 29].

According to the Digital Games Research Center, 32 million Iranians play for an average of 93 min daily. Children and adolescents aged 3 to 17 account for 42% of the participants. Children spend an average of 86 min per day gaming, whereas adolescents spend 147 min [30]. Vahidi et al. [31] reported a GD prevalence rate of 2.1% among Iranian undergraduate students. Furthermore, there was a 5.9% prevalence of IGD among Iranian primary school students, according to Areshtanab et al. [32].

To the best of the authors’ knowledge, these tools have been validated among Iranian adolescents; GAS-21 [33], GAS-7 [34], IGDS-SF9 [35], and POGQ [36]. The cut-off point for GAS-21 is unclear [37], the theoretical basis of the GAS-7 and IGDS-SF9 is DSM-5 [34, 35], and POGQ assesses problematic online gaming, which covers five out of nine criteria of DSM-5 [37].

Considering that the GD has been added to ICD-11 since 2019, A psychometric tool is needed to measure this disorder in Iran for epidemiological research and provide appropriate treatment programs. This study aims to determine the validity and reliability of the Farsi version of the Gaming Disorder Scale for Adolescents (GADIS-A) [38] in Iranian male adolescents.

## Methods

### Participants and study design

Dr. Paschke permitted the scale to be translated and normalized in Iran. The researcher translated GADIS-A items into Farsi, and one psychologist fluent in English corrected them. The translation’s authenticity was also confirmed by having these items translated back into English by a professional translator. Seven psychology professors approved the content validity of the translated scale with clinical and psychometric experience. As Haynes et al. [39] mentioned, seven experts were needed.

According to Hair et al. [40], 250 to 400 participants are needed for validity and factor analysis. By convenience sampling, 260 male Isfahan students in grades 7 to 12 were chosen to complete the GADIS-A. For convergent validity, 50 individuals were randomly chosen to fill out the problematic online gaming questionnaire (POGQ). 30 individuals were randomly selected among those who left their phone numbers. Two weeks after the first performance, they filled the scale again to assess the reliability retest.

### Measures

#### Gaming disorder scale for adolescents (GADIS-A)

Paschke et al. [38] developed this scale in 2020 to measure GD according to ICD-11 among German adolescents aged 10 to 17. It contains nine symptom items with five (Likert-scale) response options (0-strongly disagree, 1- somewhat disagree, 2-partially disagree/partially agree, 3-somewhat agree, 4-strongly agree), as well as one additional question regarding symptoms frequency with four response options (0-not at all, 1- only on single days, 2-during longer periods, 3-almost daily). The ICD-11 criteria are taken into consideration in these statements. Cognitive-behavioral symptoms (CBS), which are examined with four questions, with a cut-off point of 9, and negative consequences (NS), which are assessed with five questions with a cut-off point of 5, are the two subscales. To be diagnosed with GD, someone must score over the cut-off in both CBS and NS and pick "during longer period " or " nearly daily" in item 10. Paschke et al. [38] reported that Cronbach’s alpha for the total GADIS-A scale is 0.91, 0.9 for the NS factor subscale, and 0.87 for the CBS factor subscale. The GADIS-A has also been validated among Russian adolescents, and Cronbach’s alpha reported 0.891 [41].

#### Problematic online gaming questionnaire (POGQ)

This questionnaire was developed by Demetrovics et al. [42] in 2012, comprising 18 items. There are five (Likert-scale) response options for each question. Nazari et al. [36] validated POGQ in a sample of 360 adolescent students of Tehran and reported Cronbach’s alpha of 0.85.

### Statistical analysis

To describe the basic characteristics of the subjects, descriptive statistics were employed.

Seven experts were asked to rate the necessity of each item in Iranian culture on a three-point scale (necessary, useful but not necessary, and not necessary) to evaluate the content validity of the GADIS-A. The Lawshe [43] formula was used to determine the content validity ratio (CVR) for each item, $CVR = \frac{{ne - \frac{N}{2}}}{\frac{N}{2}}$ where Ne is the number of experts who chose "necessary" for each item and N is the total number of experts. The CVI is the average of the CVRs of the remaining items in the scale’s final version.

Internal consistency (Cronbach’s alpha) of the subscales and overall scale was used to assess reliability. According to EFPA [44], internal consistency is deemed outstanding when Cronbach’s alpha is more than 0.90, good when it is between 0.80 and 0.90, adequate when it is between 0.70 and 0.79, and insufficient when it is less than 0.70.

Pearson correlation between the GADIS-A and the POGQ was used to evaluate convergent validity, and it was also used between the GADSI-A test and retest to assess reliability. Cohen [45] classified correlation coefficients as low between 0.10 and 0.29, moderate between 0.30 and 0.49, and high between 0.50 and above.

To evaluate the two-factor model, a confirmatory factor analysis (CFA) was conducted to confirm the construct validity for ordinal categorical variables. The WLSMV should be used as a robust estimator that does not require normally distributed variables and is the best choice for modeling categorical or ordered data, given that the items were assessed on a Likert scale (ordinal scale) [46]. The WLSMV was not used, nevertheless, since Akaike’s Information Criterion (AIC) and the Bayesian Information Criterion (BIC), which are based on maximum likelihood (ML), were required for model comparison.

Consequently, ML was used as an estimator. According to Kilic et al. [47], ML can be employed as an estimator for ordinal scale when factor loadings are more than 0.7. To evaluate the two-factor model with CFA, the following goodness-of-fit indices were used: the chi-square value (χ^2^), the ratio of chi-square to the degree of freedom (χ^2^/*df*), comparative fit index (CFI), the standardized root mean squared residual (SRMR), the Tucker-Lewis Index (TLI). Root Mean-Square Error of Approximation (RMSEA). The model fit criteria were χ^2^/*df* < 2, RMSEA < 0.1 [48], SRMR < 0.08 [49], CFI > 0.9, and TLI > 0.9 [46].

CFA was carried out using the ML estimator, the R Software Packages psych [50], and lavaan [51]. SPSS version 24 was used for descriptive statistics, internal consistency, test–retest reliability, and convergent validity.

## Results

The average age of the participants was 15.1 years, with a standard deviation of 1.56 years. Table 1 displays further descriptive information about the subjects. According to the GADIS-A cut-off points, 11 participants (4.2%) meet the GD criteria, whereas 17 subjects (6.5%) complete the hazardous gaming criterion.

**Table 1:** Descriptive information of subjects

Variable	N (%)
Grade	
Seventh	34 (13.1)
Eighth	71 (27.3)
Nineth	51 (19.6)
Tenth	45 (17.3)
Eleventh	43 (16.5)
Twelveth	16 (6.2)
Age	
12	14 (5.4)
13	29 (11.2)
14	53 (20.4)
15	53 (20.4)
16	57 (21.9)
17	40 (15.4)
18	14 (5.4)
School type	
Non-profit	165 (63.5)
Governmental	46 (17.7)
Government model school	30 (11.5)
Talented	19 (7.3)
Prevalence rate	
Gaming disorder	11 (4.2)
Hazardous gaming	17 (6.5)

As seen in Table 2, Skewness and Kurtosis have absolute values of less than 3, suggesting that the frequency distribution of each item’s response is normal [40].

**Table 2:** Descriptive statistic of GADIS-A scores

Item	Mean	Standard deviation	Skewness	Kurtosis
1	2.315	1.318	− 0.241	− 1.167
2	1.473	1.345	0.642	− 0.821
3	1.061	1.244	1.046	− 0.026
4	0.938	1.099	1.177	0.660
5	1.057	1.185	1.022	0.141
6	0.942	1.118	1.165	0.525
7	0.730	1.041	1.528	1.695
8	0.669	1.053	1.652	1.932
9	1.000	1.208	1.046	− 0.041
GADIS-A	10.188	7.088	0.830	0.579
CBS	5.784	3.768	0.596	− 0.149
NS	4.403	4.096	1.169	1.381

*GADIS-A* gaming disorder scale for adolescents, *CBS* cognitive behavioral symptoms, *NS* negative consequences

### Content validity

CVI and CVR were employed to assess content validity. All ten items were deemed "necessary" by all seven experts. As a result, the value for CVI and CVR was computed to be 1, indicating that content validity is acceptable according to Lawshe [43].

### Construct validity

According to Table 3. The suitability of the items on this scale for factor analysis was evaluated using the corrected item-total correlation, and this correlation for each item was more than 0.3. indicating that each item is correlated with the total score; thus, the items are valid [52].

**Table 3:** Corrected item-total correlation

Item	Corrected item-total correlation	Item	Corrected item-total correlation	Item	Corrected item-total correlation
1	0.47	4	0.63	7	0.54
2	0.53	5	0.64	8	0.57
3	0.44	6	0.62	9	0.62

**Fig. 1:**
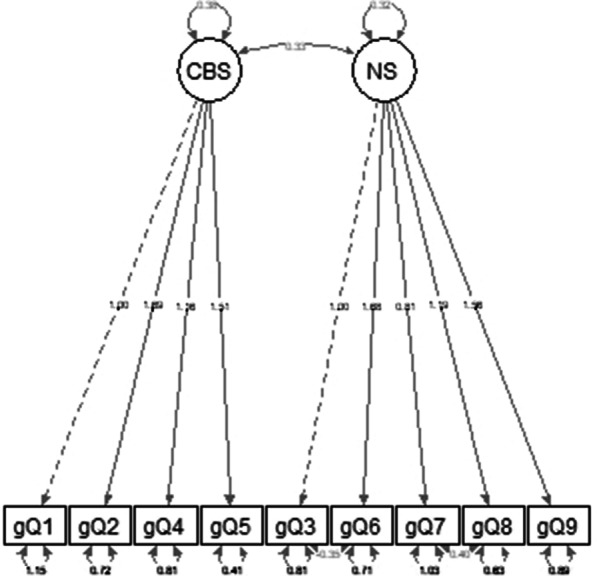
Factor loadings on two GADIS-A factors. CBS: cognitive behavioral symptoms, NS negative consequences. gQ1: GADIS-A question 1 (item 1–9)

Confirmatory factor analysis was conducted to evaluate structural validity. As shown in Table 4. Chi-square is a standard index in assessing model fitness, and its lack of significance at the level of 0.05 implies optimal model fitness [53]. Overall, the suggested fit statistics demonstrated that CFA provided a satisfactory fit; χ^2^/*df* = 1.259, RMSEA = 0.083, SRMR = 0.074, CFI = 0.934, and TLI = 0.915. Figure 1 depicts the factor loadings. In Table 5, the standardized factor loading for the CBS factor varied between 0.5 and 0.83, whereas the NS factor loadings ranged between 0.44 and 0.74. According to Table 5. the β value is positive and robust, which indicates a high correlation between the two subscales. Compared to the null model, the *p* value for the one-factor model is significant. However, The AIC and BIC for the two-factor model are lower (Table 4), indicating that the two-factor model has a better fit than the one-factor model [54].

**Table 4:** Confirmatory factor analysis of the GADIS-A

Model	χ^2^	*df*	*P* value	χ^2^/*df*	RMSEA	SRMR	CFI	TLI	AIC	BIC
One-factor	50.525	27	0.0001*	1.871	0.122	0.094	0.856	0.815	1312.2	1346.7
Two-factor	30.225	24		1.259	0.083	0.074	0.934	0.915	1298.0	1338.1

*RMSEA* Root Mean Square Error of Approximation, *SRMR* standardized root mean square residual, *CFI* Comparative Fit Index, *TLI* Tucker-Lewis index, *AIC* Akaike’s Information Criterion, *BIC* Bayesian Information Criterion

*χ^2^ significant at *p* < 0.01

**Table 5:** Factor loadings of GADIS-A

	b	t	p	β
CBS				
Item 1	1.000			0.702
Item 2	1.667	4.251	0.001	0.833
Item 4	1.725	3.918	0.001	0.715
Item 5	1.560	3.890	0.001	0.847
NS				
Item 3	1.000			0.690
Item 6	1.800	3.011	0.001	0.839
Item 7	1.416	2.827	0.001	0.688
Item 8	1.640	2.939	0.002	0.737
Item 9	2.191	3.041	0.001	0.931
Two-factor covariance	0.256	2.146	0.032	0.875

Convergent validity was evaluated using the correlation of GADIS-A scores with the POGQ. The correlation between the GADIS-A and the POGQ was 0.74, which was statistically significant (*P* < 0.001).

### Reliability

Internal consistency and retest reliability were used to evaluate the scale’s reliability. The GADIS-A has a Cronbach’s alpha of 0.85, suggesting that the scale has high internal consistency. Cronbach’s alpha of subscales cognitive-behavioral symptoms and negative consequences were 0.75 and 0.70, respectively. The scale was retested on 30 participants two weeks after the first performance to determine retest reliability. The findings had a significant correlation of 0.88 (*P* < 0.001), Indicating good retest reliability for this scale.

## Discussion

Considering the number of studies into gaming disorder around the world and the fact that GD and its criteria were recently included in the ICD-11, developing a theoretically and psychometrically instrument to evaluate GD following the new ICD-11 criteria is becoming critical. This research studied a sample of Iranian male adolescents to assess the psychometric characteristics of the GD scale for adolescents (GADIS-A). This tool includes nine GD-symptom items as well as one item measuring the frequency of GD symptoms based on the ICD-11 time criteria.

When reviewing the content validity, all seven experts agreed that all GADIS-A items were necessary. As a result, all of the items remained in the final version of the GADIS-A. This is a strength of a translated screening tool required for the subject to correctly grasp each item’s meaning and assess the clinical criteria precisely.

In this study, the problematic online gaming questionnaire was used to assess convergent validity so that increasing the score in this questionnaire leads to increasing the score of the GADIS-A. The correlation coefficient between the two instruments was 0.75, which suggests a good convergent validity.

Based on the findings, the GADIS-A is a reliable psychometric instrument for detecting GD symptoms in Iranian adolescents. The Cronbach’s alpha reliability and test–retest coefficients show the reliability of the GADIS-A and its two subscales.

The construct validity findings supported Paschke et al. [38] two-factor model. These results are congruent with Nazari et al. [41], who also discovered a two-factor structure for this instrument.

Items 1, 2, 4, and 5 are part of the first factor, referred to as cognitive-behavioral symptoms. These questions reflect an inability to control the amount of time spent playing despite the negative consequences. Research literature shows GD can cause a drop in school grades, jeopardize family, friendly and emotional relationships, and affect leisure activities [6, 55–58]. These adverse outcomes assessed in second-factor negative consequences are identified in items 3, 6, 7, 8, and 9. The scale’s final item assesses the frequency and severity of problems caused by gaming for the individual. These items are based on the ICD-11 criteria and cover all of them.

According to the Table. 1, the prevalence of GD in this research was 4.2 percent. These results are consistent with prior studies on Iranian primary school students, which discovered a 5.9 percent prevalence rate of IGD [32]. The ICD-11 has a higher diagnostic threshold for GD than the DSM-5 [59]. The similarity of the prevalence rates in these two studies is explained by the fact that the current research was conducted during the pandemic and school closure. These factors may increase the time spent playing video games [60] and the prevalence of GD among adolescents [61].

Using a large number of samples per question was one of the study’s strengths. The current research had some limitations, most notably participation and data collection. According to sampling, the convenience sample approach and the absence of female individuals limited the range of comparators for validity. There are possible cultural biases in the translating process. For example, the phrase "poor reference" in item 9 has been removed since such a reference is uncommon for admission to the university and the job market in Iran. The research was carried out during the COVID-19 pandemic and school closure, and data was collected online rather than in person. Data were acquired using self-report tools, which are prone to methodological flaws. The stressful pandemic condition may have worsened the individuals’ mental health difficulties and everyday psychological life suffering [62].

Future research should examine samples of adolescent girls because this area lacks significant research [63]. In addition, there is a major paucity of epidemiological research on GD or IGD in Iran. The current study’s standardized scale can be used in future epidemiological studies.

## Conclusion

Eventually, The gaming disorder scale for adolescents in Persian has a two-factor structure and is appropriate for use in Iran.

## Data Availability

The datasets used and/or analyzed during the current study are available from the corresponding author on reasonable request.

## Abbreviations

AIC: Akaike’s information criterion
BIC: Bayesian information criterion
CFA: Confirmatory factor analysis
CVI: Content validity index
CVR: Content validity ratio
CBS: Cognitive behavioral symptoms
DSM-5: Diagnostical and statistical manual 5th edition
EFPA: European Federation of Psychologists’ Associations
GADIS-A: Gaming disorder scale for adolescents
GAS-7: Gaming addiction scale 7 items
GAS-21: Gaming addiction scale 21 items
GD: Gaming disorder
ICD-11: International classification of disease 11th version
IGD: Internet gaming disorder
IGDS-SF9: Internet gaming disorder-short form 9 item
ML: Maximum likelihood
NC: Negative consequences
POGQ: Problematic online gaming questionnaire
WHO: World Health Organization
WLMSV: Weighted least squares mean and variance adjusted
